# The Lateral Continuity and Vertical Arrangement of Dust Layers in the Martian North Polar Cap From SHARAD Multiband Data

**DOI:** 10.1029/2022GL099896

**Published:** 2022-09-03

**Authors:** Erica R. Jawin, Bruce A. Campbell, Jennifer L. Whitten, Gareth A. Morgan

**Affiliations:** ^1^ Smithsonian Institution National Air and Space Museum Washington DC USA; ^2^ Tulane University New Orleans LA USA; ^3^ Planetary Science Institute Tucson AZ USA

**Keywords:** Mars, NPLD, Amazonian climate, resonance, SHARAD, multiband

## Abstract

Interpretation of radar sounder reflections to infer the structure and composition of the martian polar caps depends on whether bright returns correspond to single packed dust layers or a more finely layered structure. Reflections from multiple layers can create strong resonant scattering (interference) effects that impact analyses of radargram reflectors and inference of dielectric contrast. We identify resonant behavior for an areally extensive reflector in the north polar layered deposits from Shallow Radar data processed in two frequency bands. Echo strength varies by ∼2 dB between subband reflections across a region ∼400 km in extent, with the stronger echo shifting abruptly from the high‐ to low‐frequency band outside the central region of Gemina Lingula. This behavior can arise from resonant scattering between two layers of dust (0.3–0.6 m thick) separated by 0.5–3 m of ice. Such layering requires there be little postdepositional aeolian activity to preserve layer thickness and spacing.

## Key Points

- We identify resonant radar scattering behavior in radargrams of the martian north polar layered deposits using Shallow Radar multiband data
- Resonant behavior can affect interpretation of radargrams and can arise from two thin dust layers separated by less than a few meters of ice
- We characterize a reflector that formed during quiescent periods of sustained dust accumulation with minimal aeolian activity

## 1 Introduction

The martian north polar layered deposits (NPLD), composed of finely layered water ice and dust exceeding a kilometer in places, represent a record of cyclic climatic variations over the past few million years, which were likely driven by variations in orbital parameters (chiefly obliquity) (e.g., Levrard et al., 2007; Phillips et al., 2008; Putzig et al., 2009). Radar sounding data from Shallow Radar (SHARAD) and Mars Advanced Radar for Subsurface and Ionosphere Sounding (MARSIS) have revealed that the NPLD is composed of stacks of reflectors corresponding to extremely pure water ice (>95%) interspersed with thin layers of dust and/or less pure ice (e.g., Courville et al., 2021; Grima et al., 2009; Lalich et al., 2019; Nunes & Phillips, 2006). The formation of the layered structure of the NPLD is believed to be due to either (a) accumulation, where dust deposition rates are variable through time (Putzig et al., 2009); (b) ablation, where ice and dust are deposited simultaneously but periods of ice ablation develop a sublimation lag that becomes concentrated into layers (e.g., Levrard et al., 2007); or (c) a combination of (a) and (b) (Courville et al., 2021), which could operate during opposite periods of the obliquity cycle (Hvidberg et al., 2012). Individual NPLD layers therefore contain critical information about past climatic conditions on Mars, and linking layers to equivalent radar reflectors is crucial to disentangling the structure and evolution of the NPLD.

Radar sounders observe reflections from abrupt changes in the dielectric constant along a vertical cross section beneath the spacecraft. Interpreting NPLD structure is complicated by the fact that each vertical cell of a radargram corresponds to the net reflection from all physical layers within that cell, and it is not possible to distinguish echoes from a single thick dust layer or multiple thin layers. Additionally, reflections from every interface (the top and bottom of each layer(s)) will interfere with other reflections within that cell. B. A. Campbell & Morgan (2018) found variations in reflector properties in SHARAD multiband data in the NPLD, and argued that in some cases this behavior was due to resonant multilayer scattering. Similarly, Courville et al. (2021) demonstrated interference in forward models of radar sounding in multiple layers. As radargram layering is used to estimate ice and dust content, PLD composition, constrain models of PLD formation and evolution, and inform climate models (e.g., Bierson et al., 2021; Lalich et al., 2019; Smith et al., 2016), it is critical to study resonant scattering and its effect on radargrams.

We identify resonant behavior in SHARAD multiband data in the NPLD and explore potential physical implications of this behavior. Our analysis documents the scattering behavior and lateral consistency of a single reflector in Gemina Lingula across two SHARAD subbands as well as the full bandwidth (full‐BW) data. We use a 1D layer resonance model to explore the parameter space of layer thickness and separation to demonstrate a plausible scenario for the SHARAD observations, and RadSPy, an *N‐*layer forward‐modeling software (Courville et al., 2021) to examine specific physical configurations that could match our observed reflector. These modeling results and the mapped extent of the reflector are used to interpret environmental conditions during the deposition of the reflecting layer(s).

## 2 Analysis of SHARAD Multiband Data

SHARAD transmits a chirped 10‐MHz BW signal centered at 20 MHz over an 85 µs duration. After synthetic aperture processing, a SHARAD radargram has an along‐track spatial resolution of 0.3–1 km and cross‐track resolution of 3–6 km (Seu et al., 2007). The SHARAD 10‐MHz BW enables a best one‐way vertical free space resolution of 15 m. Range resolution is also modulated by the dielectric constant of the medium, $\varepsilon^{\prime }$, as 1/$\sqrt{\varepsilon^{\prime }}$, so the vertical resolution (prior to transform windowing) in water ice ($\varepsilon^{\prime }$ = 3.2) is ∼8.4 m and in solid rock ($\varepsilon^{\prime }$ = 8) is ∼5.3 m.

We generate multiband data by splitting the 10‐MHz chirped signal into separate 5‐MHz subbands. B. A. Campbell & Morgan (2018) used three subbands (“high,” “medium,” and “low” frequency) but we find that the medium band is largely redundant with the other two. In our work, we use a high‐frequency band (H, 22.5 MHz, center λ = 7.5 m in ice) and a low‐frequency band (L, 17.5 MHz, center λ = 9.6 m in ice). We apply a Hann window during range compression so the resulting vertical range resolution in ice is ∼20 m. After generating the radargrams for the H and L bands, we examined a specific reflector in both subbands and the full‐BW data and mapped its spatial distribution using JMars (Christensen et al., 2009). Differences in the ratio of band power in H and L due to variations in SHARAD transmitted power over the BW are compensated during radar processing, using a calibration factor based on a large sample of SHARAD surface echoes across Mars.

**Figure 1:**
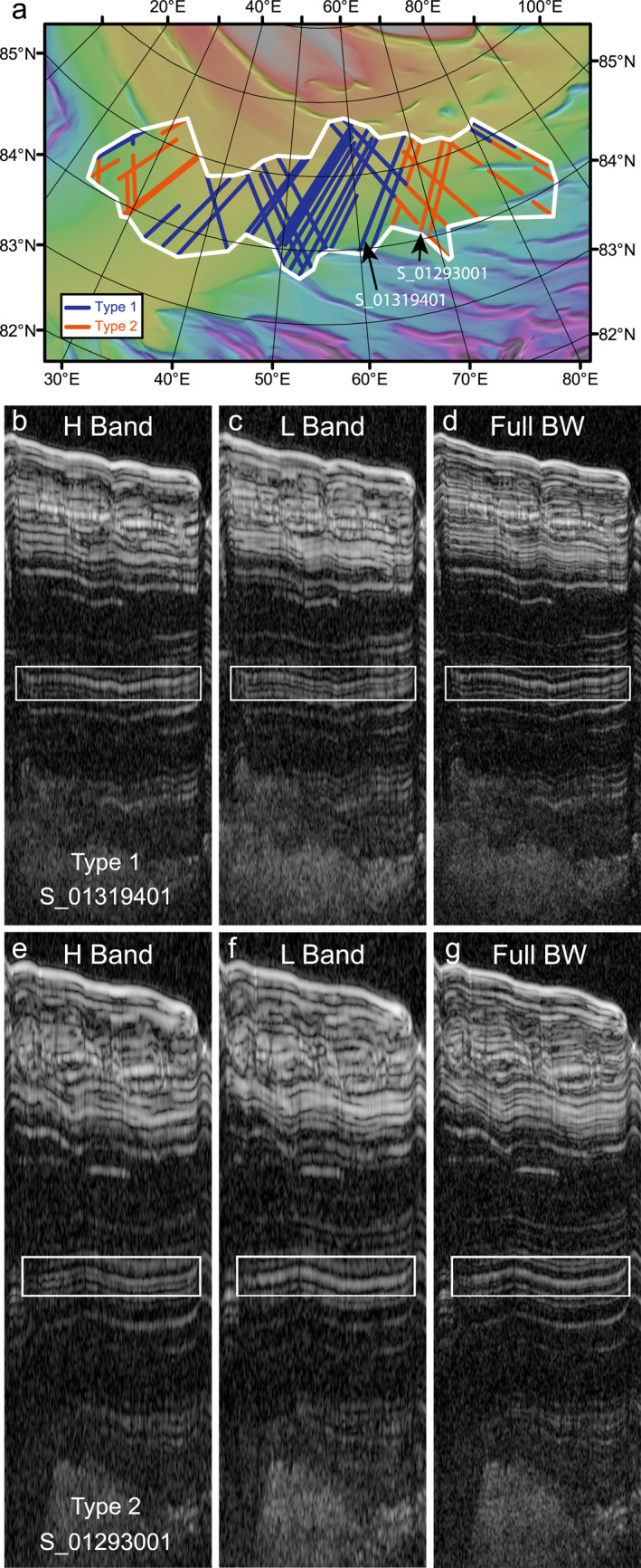
(a) Distribution of the resonant reflector in Gemina Lingula. Blue lines are portions of Shallow Radar (SHARAD) tracks showing Type 1 behavior, red lines are Type 2. White region encloses all mapped Type 1 and 2 behaviors. (b–d) Portion of SHARAD track S_01319401. (e–g) Portion of track S_01293001. Boxed region shows the variable reflector properties in the subbands and full‐BW. Shaded basemap is MOLA elevation ranging from −4 km (purple) to −2.5 km (white).

Our analysis focuses on a single reflector in the Gemina Lingula region (Figure 1a), the fourth up from the bottom of Unit F of Putzig et al. (2009). This reflector changes in appearance between the H and L band. In track S_01319401, it is bright in the H band but much dimmer in the L band (Figures 1b and 1c). A second, dimmer reflector obvious only in the L band makes it easier to trace the reflector across radargrams but is not part of our modeling effort below. Other tracks, however, show the reverse behavior: a brighter reflection in the L band than in H (Figures 1e–1f). We refer to the former behavior (brighter in H) as Type 1 behavior and the reverse (brighter in L) as Type 2. In Type 2 behavior, the second dim reflector is more evident in the H band rather than L.

**Figure 2:**
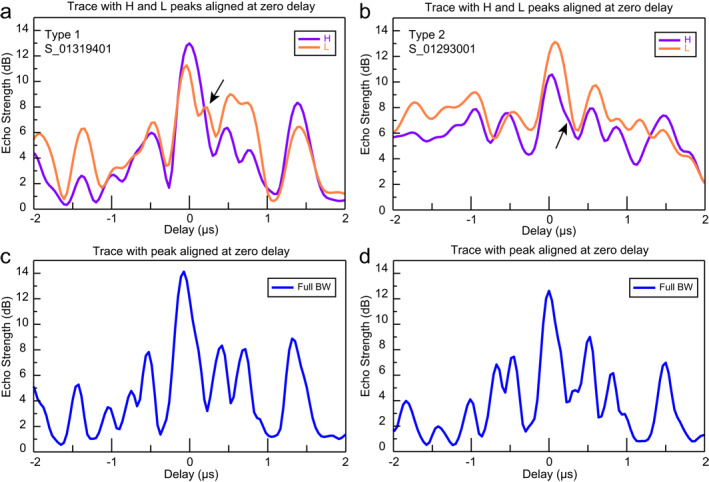
Plots of power for the reflector traced in track (a) S_01319401, Type 1 and (b) S_01293001, Type 2. Power was averaged along‐track for a portion of the radargram (e.g., white boxed region, Figures 1b and 1e), and the peaks in the H and L bands were aligned at an arbitrary zero delay. The reflector shows a broad peak at higher power (a) in H (purple curve) and (b) in L (orange curve) compared to a smaller peak (a) in L and (b) in H. Note the presence of the second smaller peak (a) in L and (b) in H (arrows). (c–d) The same region in the full‐BW radargrams.

We quantified the change in echo power of the reflector in both bands by tracing its extent along a segment of the radargram (boxed regions in Figure 1) and averaging power along the track. The changes in brightness in Figures 1b to 1c, and 1e to 1f represent a ∼2.0 dB difference in peak echo power for both Type 1 and 2 behaviors (stdev: ∼1.5 dB along‐track) (Figure 2). The second reflector is resolvable as a peak 3 dB weaker (Figure 2, arrows).

We confidently identified this reflector in 36 SHARAD tracks (Figure 1a, Table S1 in Supporting Information S1). Twenty‐six tracks exhibit Type 1 behavior, while 16 show Type 2. Additionally, 6 tracks contain both Type 1 and 2 behaviors at different locations (Figures 1a, and Table S1 in Supporting Information S1). The distribution of this reflectsor across Gemina Lingula (Figure 1a) spans ∼400 km and covers a region several tens of thousands of km^2^ and the distribution of each Type is spatially coherent. Type 1 behavior is concentrated in the central portion of Gemina Lingula, contiguous over ∼200 km and in small distal portions of the analysis region (Figure 1a). Type 2 behavior is dominant on the eastern and western portions. Transitions between the Types are consistent across several intersecting tracks. Transitions along the same track occur abruptly over ∼1 km (∼2 SHARAD samples) with no apparent change in elevation or slope to explain the transition (Figure S1 in Supporting Information S1). All the analyzed tracks inside the white region of Figure 1a show evidence of either Type 1 or 2 behavior in the reflector. Note that we only outlined portions of tracks in Figure 1a where we could confidently identify the reflector. The full extent of the reflector could be larger than what is presented.

In the full‐bandwidth SHARAD data, there is no apparent difference between tracks with Type 1 or Type 2 behavior in the multiband data. The reflector does not consistently resemble the properties of the H or L band, although it remains brighter than the surrounding reflectors (e.g., Figures 1d and 1g). The second dim reflector most often cannot be confidently resolved in the full‐BW data. The peak echo strength of the reflector in the full‐BW data is on average not significantly different from the multiband data (1 dB less, within one stdev) (e.g., Figure 2d) but in individual tracks it can be up to 4 dB weaker in the full‐BW data—illustrating how resonant scattering has a variable effect on reflector appearance.

## 3 Two‐Layer Resonant Echo Models

The fact that we see the Type 1 and 2 behaviors in a single SHARAD reflector, and that these behaviors persist over large regions, suggests that there must be a multilayer resonant scattering phenomenon to explain the change in band power, and the structure of these layers must be relatively uniform in order to preserve the resonant behavior. We implement two models of layered ice and dust to identify a simple structure that could give rise to resonant scattering, similar to our observations. Note that it is not possible to find a single unique configuration of layer(s) that match our observations. We instead implement one‐ and two‐layer models over a range of layer thickness and spacings that are (a) physically plausible, meaning that they adhere to expected ice/dust concentrations of the NPLD as a whole, and are (b) resolvable in multiband data, such that the total thickness of all layers remains within a single resolution cell. For each simulation, we calculate the echo power in the H and L bands and take their ratio in dB, which we refer to as *H/L*. Type 1 behavior corresponds to *H/L* > 0 dB while Type 2 behavior has *H/L* < 0 dB, and values approach zero dB (unity) where power is equal in both bands. We attempt to find a simple model that resembles our observations, where *H/L* takes on strongly positive and negative values without a significant intermediate population of *H/L* ∼ 0 dB.

We assume a matrix of ice with a dielectric constant of $\varepsilon_{1}^{\prime }$ = 3.2 containing two embedded layers of packed dust with $\varepsilon_{2}^{\prime }$ = 4.5 (although results are not qualitatively different if $\varepsilon_{2}^{\prime }$ = 6). The thickness of the two dust layers is given by *d*_1_ and *d*_2_, and their separation is *s*. *H/L* is a function of these parameters and the radar wavelength, λ. At a single frequency of the chirp, *f*, the wavelength in a medium is as follows:

$$\lambda \left(\varepsilon^{\prime },f\right)=\frac{c}{f\sqrt{\varepsilon^{\prime }}}$$

where *c* is the speed of light in vacuum and $\varepsilon^{\prime }$ is the dielectric permittivity of the medium. The *H* and *L* band wavelengths (in dust with $\varepsilon^{\prime }$ = 4.5) are therefore 6.28 m (H) and 8.08 m (L), respectively. If we trace the round‐trip phase of the four echoes (the top and bottom of the two layers), the net echo amplitude at each value of *f* is their phasor sum which considers interference between the returning signals. Constructive interference occurs when signals are half a wavelength apart, and destructive interference occurs at a quarter wavelength. However, because the SHARAD signal from a multiband channel spans a range of frequencies, the actual behavior is blurred from a simple resonant pattern cycling between perfect constructive and perfect destructive interference, and we must integrate the reflected power to obtain the actual behavior (Lalich et al., 2019). The total modeled power in each subband is thus the integral over all the frequencies as filtered by the Hann window.

Since the reflection coefficient is the same at every interface, we can write the phase (in radians) relative to the top of the first dust layer (note that this does not consider multiple reflections between interfaces):

$$\phi_{0}=0$$

$$\phi_{1}=\frac{4\pi f}{c}d_{1}\sqrt{\varepsilon_{2}^{\prime }}$$

$$\phi_{2}=\phi_{1}+\frac{4\pi fs\sqrt{\varepsilon_{1}^{\prime }}}{c}=\frac{4\pi f}{c}\left[d_{1}\sqrt{\varepsilon_{2}^{\prime }}+s\sqrt{\varepsilon_{1}^{\prime }}\right]$$

$$\phi_{3}=\phi_{2}+\frac{4\pi fd_{2}\sqrt{\varepsilon_{2}^{\prime }}}{c}=\frac{4\pi f}{c}\left[d_{1}\sqrt{\varepsilon_{2}^{\prime }}+s\sqrt{\varepsilon_{1}^{\prime }}+d_{2}\sqrt{\varepsilon_{2}^{\prime }}\right]$$

and add the four complex phasor terms $\left(\cos\phi_{n},-\sin\phi_{n}\right)$ before squaring the amplitude to derive a reflected power at *f.* Pairs of layers near another interface may create more interference effects (Courville et al., 2021); however, our first‐order approach treats the two dust layers in isolation.

We ran this model to calculate the band power ratio, *H/L*, for a large number of randomized values of *d*_1_, *d*_2_, and *s*. One‐layer models at reasonable layer thicknesses (30 cm–5 m) strongly favor negative *H/L* values (Figure S2 in Supporting Information S1), so we implemented a two‐layer model that can more closely match our observations of bimodal *H* */* *L* behavior.

For two‐layer configurations we vary dust layer thicknesses over 30–60 cm and layer separation, *s*, over 0.5–4.0 m. Layer thicknesses were selected based on the number of reflectors within the NPLD, the need to keep the total dust abundance below ∼5% (Grima et al., 2009), estimates of surface lag thickness, and reflection models of Courville et al. (2021).

**Figure 3:**
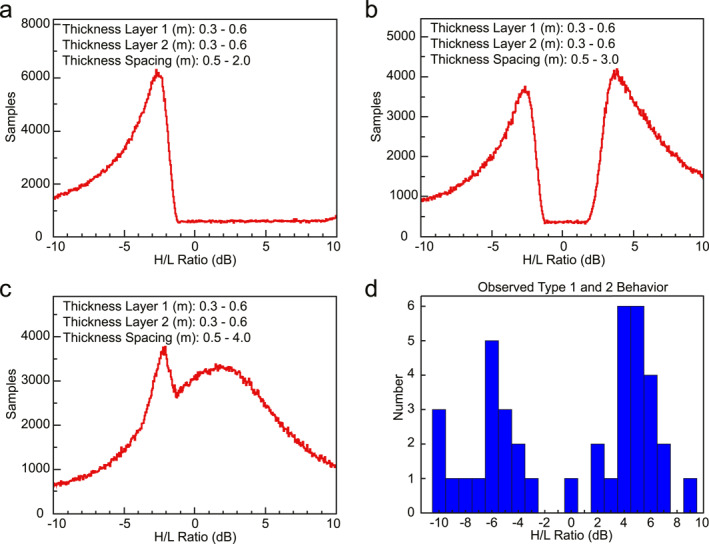
Plots of the ratio of power in H and L bands (*H/L*), for two‐layer models with 30–60 cm layers and separations from 50 cm up to (a) 2 m, (b) 3 m, and (c) 4 m. The dielectric constant of the dust layers is 4.5 and ice is 3.2. (d) *H/L* calculated from 36 SHARAD tracks of Figure 1a.

The two‐layer model results suggest that layer separation largely controls the distribution of *H/L* (Figures 3a–3c). If the separation of the two layers ranges up to 2 m, there is a single dominant peak at *H/L* < 0 dB (Type 2 behavior) (Figure 3a). When separations up to 3 m are permitted, a second peak appears in the distribution at *H/L* > 0 dB (Type 1 behavior) (Figure 3b). If the range is extended to 4 m, however, these two modes merge into a single distribution (Figure 3c). The modeled distribution of Figure 3b is bimodal and closely matches the observed reflector *H/L* values from SHARAD data (Figure 3d), with peaks at about ±5 dB. Note that our model may not capture all ranges of layer thickness and spacing that create resonant scattering but shows that a modestly constrained scenario can replicate observed Type 1 and Type 2 behaviors.

**Figure 4:**
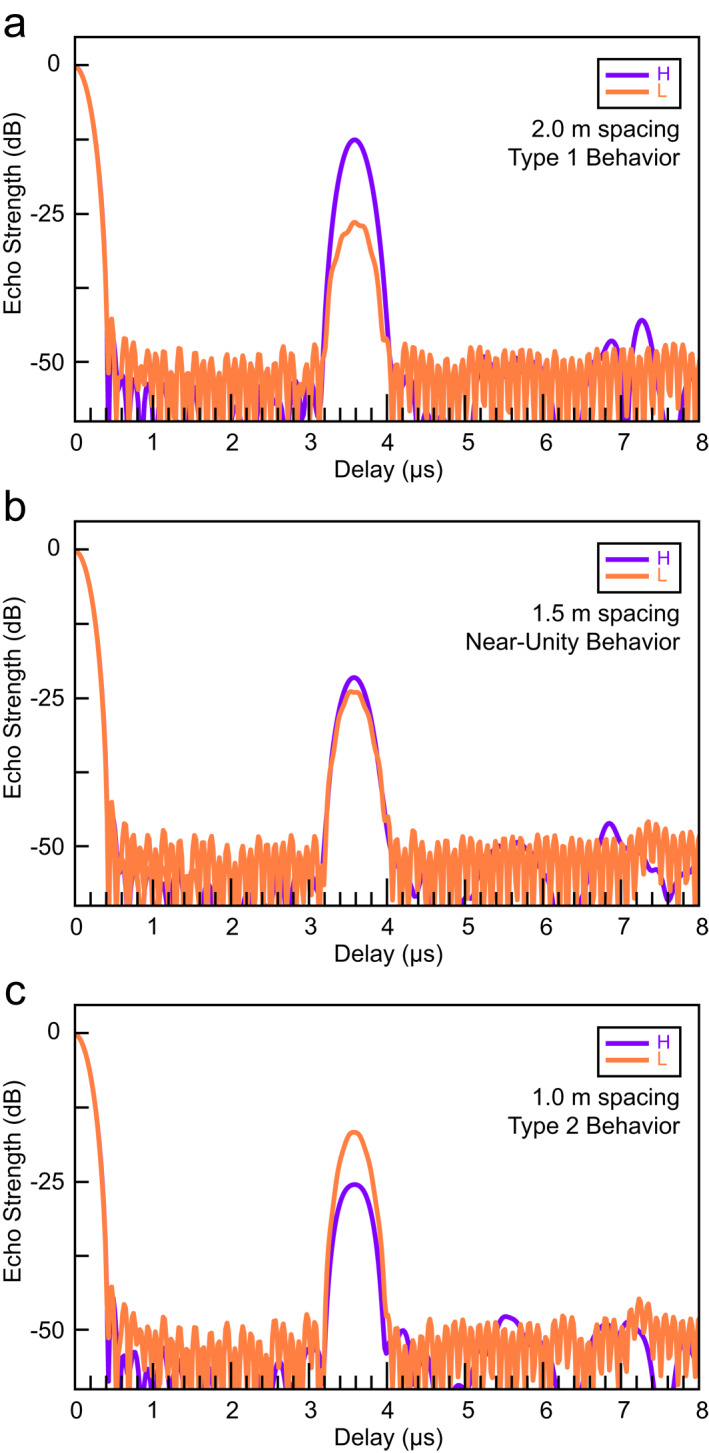
*N*‐layer radar sounder forward modeling results. Two‐layer models at layer separations (a) 2 m, (b) 1.5 m, (c) 1.0 m, showing variable echo power that replicates (a) Type 1 and (c) Type 2 returns. Certain layer separations result in similar echo strength in both bands (b). All models include a 300 m‐thick ice layer (to avoid strong surface return sidelobes) overlying two 0.5 m thick dust layers.

As an additional investigation into layer interference, we use the open source *N*‐layer radar sounder modeling software RadSPy (https://github.com/scourvil/RadSPy.git) (Courville et al., 2021), which solves the 1D *N‐*layer reflectance problem for waves in a layered medium (Wait, 1970) for the ideal SHARAD source pulse and matching filter. We model radar echoes in various configurations of two packed dust layers 50 cm thick in ice of variable thickness (dust $\varepsilon^{\prime }$ = 4.5, ice $\varepsilon^{\prime }$ = 3.2) with loss tangents of 0.01 for dust (B. A. Campbell & Morgan, 2018) and 0.001 for ice (Grima et al., 2009). Certain separations show the H band echo power is stronger than L (Figure 4a), while others show L stronger than H (Figure 4c), replicating the Type 1 and 2 behaviors respectively. Relatively few simulations show minimal separation between the bands (Figure 4b).

We also use the *N‐*layer forward model to randomly assess the same parameter space as the model from the preceding section (Figure 3) and find similar distributions of *H/L* (Figure S4 in Supporting Information S1). Our results confirm the bimodal distribution of two‐layer configurations with random layer spacings from 0.5 to 3.0 m. In fact, both the 1D layer resonance model and the *N‐*layer forward model show that the peaks of the bimodal *H/L* distribution (Figures S3b and S4b in Supporting Information S1) are generated at different layer spacings: the left‐hand peak of the distribution (*H/L* < 0, Type 2) arises from layer spacings of 0.5–2.0 m (Figures S3a and S4a in Supporting Information S1), which is anticipated due to more rapid onset of destructive interference in the H band due to its shorter wavelength. The right‐hand peak of the distribution (*H/L* > 0, Type 1) arises from layer spacings of 2–3 m (Figure 4c in Supporting Information S1)—these spacings span both the half wavelength of the H band (leading to constructive interference) and the quarter wavelength of the L band (destructive interference).

## 4 Discussion

The primary result of the modeling in Section 3 is that a modestly constrained suite of layer parameters (thickness 30–60 cm and separations 0.5–3.0 m) for a two‐layer case can yield a bimodal distribution of *H/L* and that minor perturbations do not cause an abrupt shift away from the two dominant modes. The range of plausible layer configurations is consistent with previous SHARAD modeling results (Courville et al., 2021), as well as analyses of HiRISE images which found layers ∼tens of cm thick exposed in troughs in the NPLD (Herkenhoff et al., 2007).

If we assume that our model results of two thin (∼0.5 m) dust layers separated by 0.5–3 m of ice reflect the physical structure of the mapped reflector in the NPLD, what were the climatic conditions under which this layering formed? The lateral continuity of the reflector (Figure 1a) implies that these layers extend for several hundreds of km with minimal variation in separation (the dominant factor influencing resonant behavior). Type 1 behavior may arise from random layer separations of 2–3 m and is concentrated in the central portion of Gemina Lingula. Type 2 behavior appears concentrated toward the eastern and western edges of the mapped region (Figure 1a) and may arise from layer spacings of 0.5–2 m. As such, the Type 1 and Type 2 regions may reflect up to a factor of ∼2 difference in net ice accumulation rate, with higher rates concentrated at the center of Gemina Lingula.

Two dominant models of dust layer formation are debated: Periods of enhanced dust deposition and enhanced ice ablation (i.e., formation of sublimation lag). Hvidberg et al. (2012) suggest that layers formed through dust deposition are often broad and characterized by multiple thin layers. Given the broad distribution of the Type 1 and 2 patterns and our modeling results, dust deposition is a plausible formation mechanism. If so, we can use the Hvidberg et al. (2012) upper limits on dust deposition rate of ∼0.02 mm/yr and ice accumulation rate of 0.5 mm/yr. Two dust layers ∼30–60 cm thick would require ∼15–30 kyr each of constant deposition (plus ∼25–150 kyr to deposit the ∼0.5–3.0 m of ice between the layers, although this estimate does not consider variations in ice accumulation rates in the Type 1 vs. Type 2 regions discussed above).

Layer formation via ice ablation and accumulation of sublimation lag is also feasible. In fact, the ablation model may more easily match observations, as this model could potentially provide more room for environmental variations across our broad study area. Modeling of insolation‐induced sublimation in the polar spiral troughs (Bramson et al., 2019) predicts rapid lag deposit formation, several tens of cm thick, within only 1,000 years at obliquities of ∼30–40°. Deposits on this scale are sufficiently thick to prevent further sublimation (Bramson et al., 2019), which could allow broad regions to evolve lag deposits without requiring uniform lag formation rates across such a large area.

The mapped reflector extends laterally for several hundred kilometers, so significant postdepositional reworking or lateral transport of material could not have occurred. This would require quiescent climatic periods of ∼15–30 kyr if the layers are formed via the dust accumulation model. While the ablation model formation time is shorter by up to an order of magnitude, the same requirement applies: Once formed, the sublimation lag must have remained at a relatively uniform thickness and cannot have experienced extensive reworking or lateral transport, implying a low‐energy surface environment. Long‐duration quiescent periods over such a large portion of the north pole of Mars implies a very different aeolian setting to that of the current north polar region, which is characterized by persistent katabatic winds (e.g., Spiga & Smith, 2018).

## 5 Conclusions

Our analysis documents resonant (frequency‐dependent) radar scattering within a single SHARAD reflector extending for several hundred km in Gemina Lingula, which modeling suggests may be attributed to two closely spaced (0.5–3.0 m), thin (30–60 cm) dust layers within the NPLD. Estimates of layer thickness and separation correspond to total duration for emplacement of about 50–200 kyr based on dust deposition models and much shorter timeframes if the dust‐rich layers are a sublimation lag. In either case, aeolian activity during and immediately following deposition must have been minimal in order to preserve the layering across hundreds of km.

While we discuss a single SHARAD reflector in this work, preliminary investigations suggest resonant behavior is common in the NPLD. Given the widespread observations of nonunity subband power ratios and a variable number of reflectors being resolved in multiband and full‐bandwidth SHARAD data (e.g., Figure 1), the full‐bandwidth echoes may be a poor proxy for the dielectric contrast (and thus dust content) of a single dust layer in the PLD. Resonant behavior is also likely within MARSIS data, albeit at different vertical spatial scales from SHARAD given the different radar frequencies. Looking beyond Mars, the role of resonant behavior in icy targets in sounding radar data is highly relevant for RIME and REASON studies of the Galilean satellites.

## Supporting information

Supporting Information S1

## Data Availability

SHARAD full bandwidth data are available on the PDS. Multiband data used in this work are available on FigShare (B. Campbell, 2020). RadSPy is open source software and is available at https://github.com/scourvil/RadSPy.git. JMars is free and available for download at https://jmars.asu.edu/download.
