# Supplementary material for: The Lateral Continuity and Vertical Arrangement of Dust Layers in the Martian North Polar Cap From SHARAD Multiband Data
Source: Geophys Res Lett. 2022 Sep 3;49(17):e2022GL099896. doi: 10.1029/2022GL099896 (PMC9542121; doi:10.1029/2022GL099896)
Supplement: Supplementary file 1 — Supporting Information S1 [file GRL-49-e2022GL099896-s001.pdf]

AGU PUBLICATIONS

Geophysical Research Letters

Supporting Information for

The lateral continuity and vertical arrangement of dust layers in the martian north polar cap from SHARAD multiband data

Erica R. Jawin[1], Bruce A. Campbell[1], Jennifer L. Whitten[2], Gareth A. Morgan[3]

[1] Smithsonian Institution, National Air and Space Museum, Washington, DC
[2] Tulane University, New Orleans, LA
[3] Planetary Science Institute, Tucson, AZ

Contents of this file

Table S1
Figures S1 to S4

Track	Type 1 behavior	Type 2 behavior
266002		x
393802	x	
436002	x	x
557402		x
598201	x	x
599501	x	
619302	x	
1232402	x	x
1293001		x
1319401	x	
2192701	x	x
2222801	x	x
2225901	x	
2227001		x
2262302	x	*x*
2273201	x	x
2276001		x
2303202		x
2334001		x
2402401		x
3179201	x	x
3195801		x
3202501	x	x
3210901		x
3240701	x	
3263101	x	
4017401	x	
4100801	x	x
4728101	x	x
4730802	x	
4758401		x
5622202	x	
2303601	x	x
3825302	x	
3224001	x	x
4099602	x	

Table S1. List of observed SHARAD tracks and whether Type 1 or 2 behavior is present (or both)

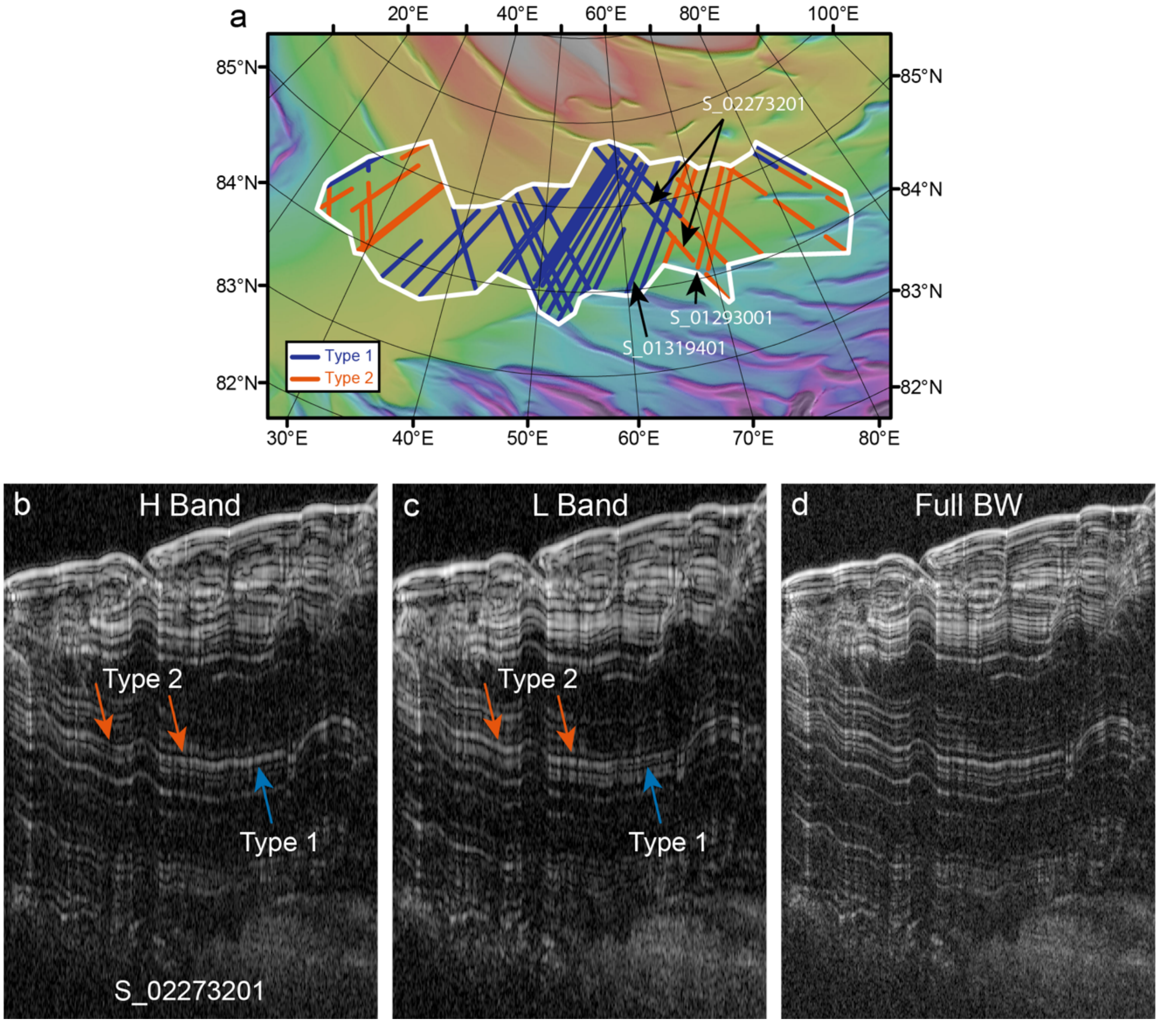

Figure S1. Example of a SHARAD track with a transition from Type 1 to 2 behavior (compare blue and red arrows in H and L bands, respectively). The transition occurs abruptly over ~2 SHARAD samples, a distance of approximately 1 km. (a) Same as Fig. 1a. (b) Track S_02273201.

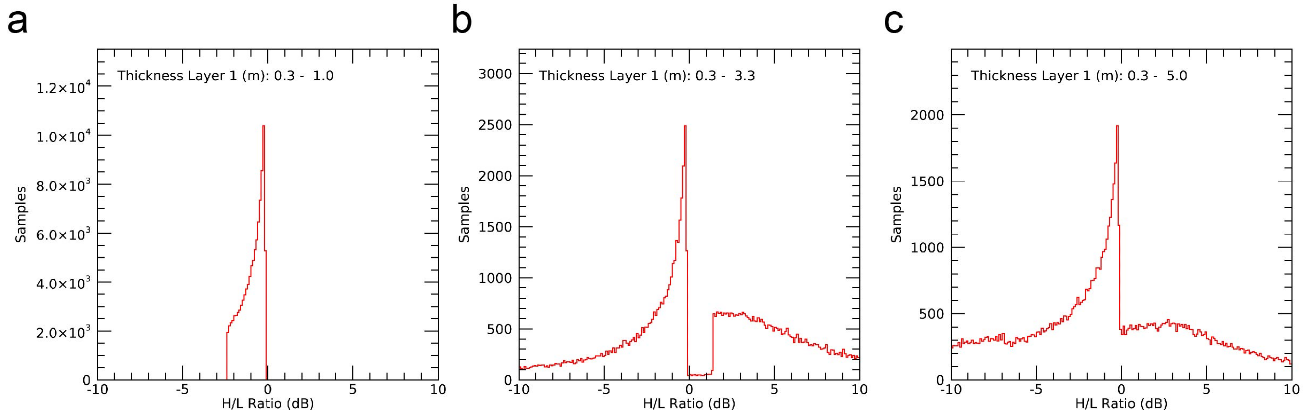

Figure S2. Statistical distribution of the ratio in echo power between H and L bands (*H/L*) in SHARAD data for a collection of single layers with random thickness between 30 cm and 5 m. The dielectric constant of the dust layer is set to 4.5. (a) The distribution is unimodal, with a slight offset to negative values corresponding to lower H band values, as the shorter wavelength of the H band (relative to L) leads to a more rapid onset of destructive interference. (b) Positive values of *H/L* are only expected once the thickness includes the H band half-wavelength (constructive interference) thickness of ~3 m, resulting in a small second peak at *H/L* > 0. (d) The general shape of the distribution does not change significantly if the thickness is increased up to 5.0 m. Thicknesses larger than 5 m are not included as this approaches the SHARAD vertical resolution. We find that while the one-layer model can generate a bimodal distribution of *H/L*, a reasonable range of thicknesses cannot reproduce a strongly bimodal distribution where negative *H/L* values (equivalent to observed Type 2 behavior) are approximately as common as positive *H/L* values (Type 1 behavior). We therefore favor the two-layer model to explain our observations, although resonant scattering within a single layer is certainly feasible in the NPLD.

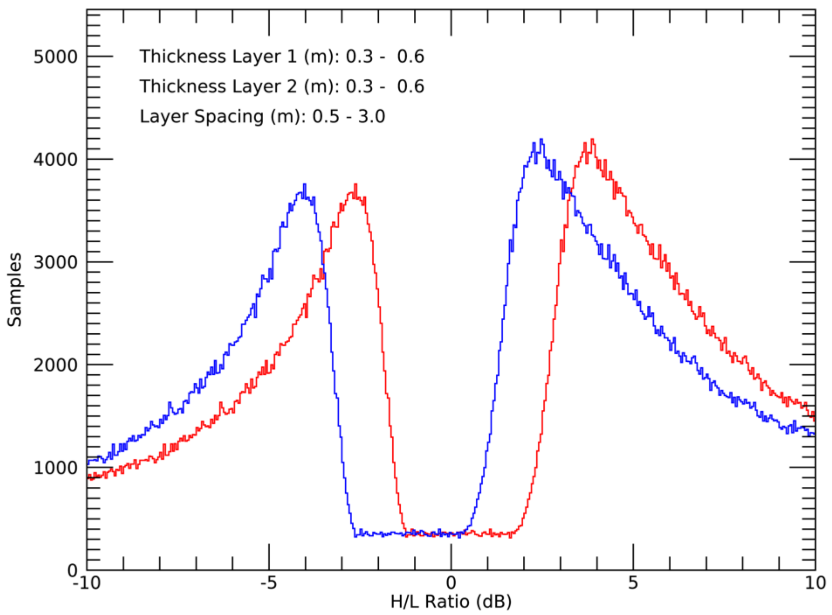

Figure S3. Proposed range of model parameters consistent with the Type 1 and Type 2 reflections when accounting for attenuation. Red plot shows no differential attenuation with depth; blue plot shows results for layers at 10 μs round-trip delay when the bulk loss tangent of the NPLD is 0.001. As the attenuation difference between the two sub-bands (not the overall loss of the full-bandwidth echo) increases with depth in the PLD, the histograms in these plots will move to the left, which can lead to changes in the signatures we associate with two-layer effects. For a sense of this issue, we can calculate the loss difference based on the loss tangent, tanδ, of the NPLD for different depths, using the model from Campbell & Morgan (2018):

$$\frac{P_H}{P_L} = exp[-31.5\ t\ tan\delta]$$

where *t* is the round-trip delay in μs. If we take the upper limit of NPLD loss tangent as 0.003 (Grima et al., 2009), then the maximum change in the band-pass power ratio is ~10 dB at 24 μs (2000 m depth for ε'=3.2). Our study layers in the NPLD are at time delay of ~10 μs, so we can estimate a maximum 4-dB leftward shift of the red histogram in the figure. If the loss tangent is 0.001, then that offset declines to only 1.4 dB (blue histogram). The latter value is more consistent with the non-unity ratios for Type 1 and Type 2 reflection patterns.

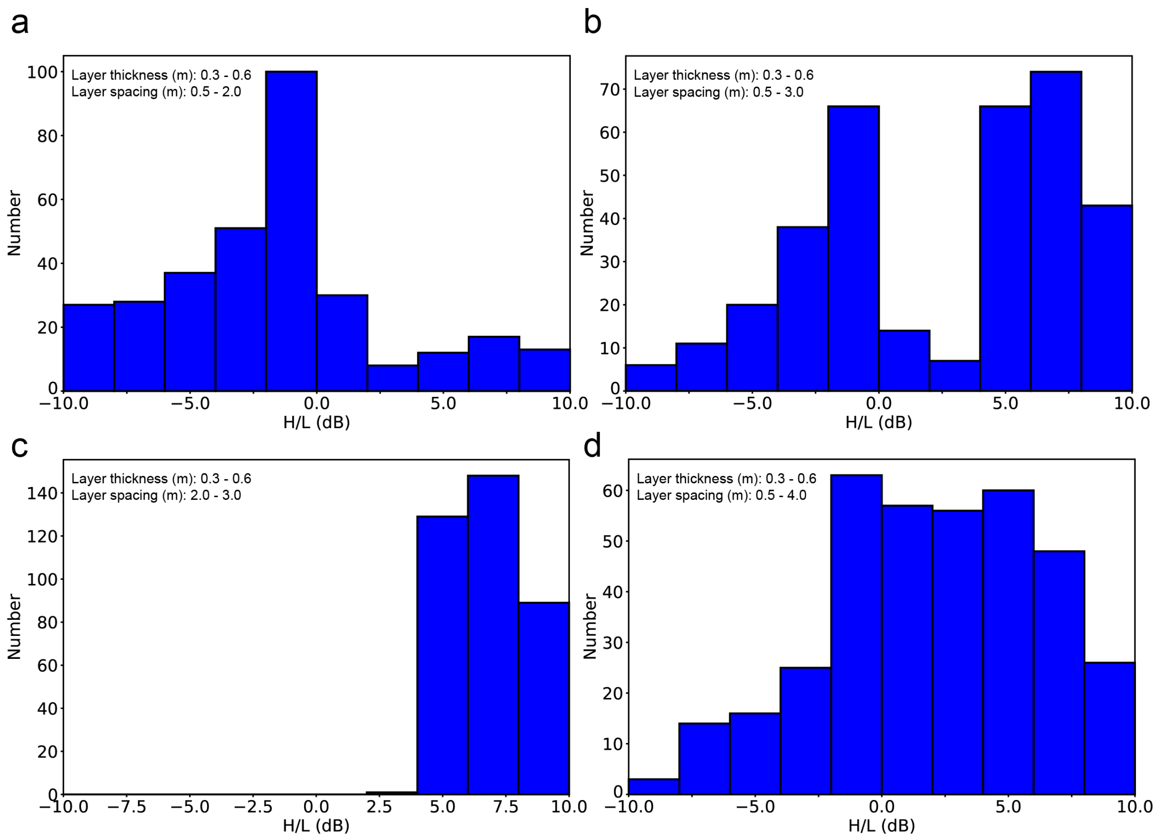

Figure S4. Statistical distribution of P_H/P_L from the *N*-layer forward model, using random pulls from the same range of layer spacing and thickness as in **Fig. 3a-c** with the 1D layer resonance model. The bimodal distribution in (b) appears to be a combination of layer spacings in two different regimes; the left-hand peak of the distribution (negative *H/L* values, corresponding to Type 2 behavior) appears to arise from random spacings between 0.5 m and 2.0 m (a), while the right-hand portion of the distribution (positive *H/L* values, Type 1 behavior) arise from random spacings only between 2 and 3 m (c). (d) At spacings up to 4 m the bimodal distribution disappears.
